# Particularities of psychiatric disorders among healthcare professionals: about a Tunisian study

**DOI:** 10.1192/j.eurpsy.2026.11300

**Published:** 2026-07-31

**Authors:** W. Ayed, M. Mersni, S. chemingui, Y. Sellaouti, G. Bahri, N. Houssem, M. Bani, N. Ladhari

**Affiliations:** 1 Occupational Medicine Department, Charles Nicolle Hopsital, Tunis; 2 Psychiatric G, Razi Hospital, Mannouba, Tunisia

## Abstract

**Introduction:**

Healthcare professionals (HCPs) are exposed to intense occupational stressors that increase their vulnerability to psychiatric disorders.

**Objectives:**

- To describe the socio-professional characteristics of HCPs suffering from psychiatric disorders
- To Specify the impact of psychiatric disorders on the medical fitness for work.

**Methods:**

Retrospective descriptive study of HCPs who consulted the occupational pathology and fitness for work department, during the period from August 2022 to September 2025, as part of a medical examination for long-term absence.

**Results:**

Of the 2840 consultants who presented for medical control, 2404 (84,6%) were HCPs. The average age was 45,9 ± 15,7 years. They were predominantly female, with a sex ratio (M/F) of 0,24. The most occupied work positions were : nurses (30,6%), workers (29,2%) and senior technicians (19,3%). They belonged to the medical (76,8%) and surgical services (23,2%). The most common psychiatric disorders were: depression (82,8%), bipolar (5%), and anxiety disorders (5%). The average length of time off work was 62±44 days. The factors contributing to the decompensation of psychiatric disorders were were social in nature (36,3%), work-related (26,8%), and related to a chronic pathologies (36,1%). Medical decisions were to justify sick leave (85%) and return to work (15%).

**Conclusions:**

Our study underscore a substantial burden of psychiatric morbidity among Tunisian HCPs and highlight the interplay of social, medical and occupational determinants. A systematic mental-health screening in occupational settings, early multidisciplinary management (occupational medicine, psychiatry, social services) and targeted workplace interventions is indicated.

**Disclosure of Interest:**

None Declared

